# 

**Published:** 1901-05-04

**Authors:** 


**Notices of Births, Deaths, and Marriages** are inserted in this column at a charge of 2s. 6d. for an announcement not exceeding four lines.

**DEATH.**

In London, on April 19th, Georgina M. E. Blackett, late masseuse, Winchester, elder daughter of J. S. Blackett, Aberfoyle, Scotland, aged 34. (1175)

### NOTICE TO CORRESPONDENTS.

**All MSS., Letters, Books for Review, and other matters intended for the Editor should be addressed THE EDITOR, "THE HOSPITAL," THE HOSPITAL BUILDING, 28 & 29 SOUTHAMPTON STREET, STRAND, W.C.**

**The Editor cannot undertake to return rejected MS., even when accompanied by stamped directed envelope.**

**Notice to Advertisers and Subscribers.**

**All Advertisements, Orders for Copies of THE HOSPITAL, and Business Communications should be addressed to THE MANAGER (Not to the Editor), THE HOSPITAL, 28 & 29 Southampton Street, Strand, London, W.C.**

**The Cost of Subscription, post free, is as follows:—Per week, 2½d.; per quarter, 2s. 8d.; per half-year, 5s. 3d.; per annum, 10s. 6d. Foreign:—Per annum, 15s. 2d., payable, in all cases, in advance.**

**NOTICE.—Vol. XXIX. of "The Hospital" (October 1900, to March, 1901), in handsome cloth binding will shortly be on sale at the Office of this Journal, price, 7s. 6d. Cases for binding the Numbers contained in this Volume 1s. 6d. Index to Vol. XXIX., 2½d., post free.**

**The Monthly Part for May is now ready. Price 6d., by post 8d.**

### SCRAPS AND GLEANINGS.

DURING the past year the working men of Belfast contributed the sum of £2,700 to the fund of the Belfast Royal Hospital.

A SUM of £382 is still needed to complete the amount of £20,219 expended on the reconstruction and enlargement of the Burton Infirmary.

A BAZAAR was recently held at Mexborough for the purpose of raising £500 in order to erect a new hospital to replace the existing Montagu Cottage Hospital.

DURING the past year the War Office sent 35 soldiers to the home of the Newcastle Convalescent Society, and two anonymous friends of the society had supplied funds for the benefit of these soldiers.

THE number of in-patients admitted during the past year to the Sussex County Hospital was 1,746. Of these 610 were discharged cured, and 119 died, 20 of which deaths occurred within 24 hours of the patients' admission.

DURING 1900, 94 soldiers were received at the Combe Down Convalescent Home, Bath, whose average stay was five or six weeks. With very few exceptions all the cases were greatly benefited, many of the men being quite fit to return to duty. Those received, included men from Australia, Canada, and South Africa.

THE new operating rooms and accessories at the Sussex County Hospital cost £3,000; the cost of the remodelling and refurnishing of the laundry has cost upwards of £3,000. A further sum of £3,000 is still required for building operations in the shape of two sanitary towers which the committee urgently recommend should be built.

AT the recent annual meeting of the Sussex County Hospital a new statute was added to the laws providing that no person shall hold office after the age of 65 years, or for more than 20 years, from the date of his election on the staff. A motion to grant two beds to every physician on his retirement, if elected consulting physician, was rejected.

AT a recent meeting at the Guildhall, Bath, called to promote the effort to raise £700 in order to continue the Convalescent Home for Wounded Soldiers on Combe Down for another year, General Ian Hamilton, who was the chief speaker, gave a description of the splendid conduct of the men at the fights of Elandslaagte, Rietfontein, and Waggon Hill, where he served with the Devons and Gloucesters. He spoke in high praise of them, and said that the British soldier was as kindly as he was brave; and he appealed to the people at home to provide the wherewithal to enable such convalescent homes as that on Combe Down to be continued for the benefit of these men. The result of the meeting was a sum of £250 collected and promised.

28 & 29 SOUTHAMPTON STREET, STRAND, W.C.

